# RNA-Seq Analysis Revealed circRNAs Associated with Resveratrol-Induced Apoptosis of Porcine Ovarian Granulosa Cells

**DOI:** 10.3390/cells13181571

**Published:** 2024-09-19

**Authors:** Huibin Zhang, Haibo Ye, Hanyu Zhou, Yangguang Liu, Fan Xie, Qianqian Wang, Zongjun Yin, Xiaodong Zhang

**Affiliations:** 1 College of Animal Science and Technology, Anhui Agricultural University, Hefei 230036, China; zhanghuibin1997@126.com (H.Z.); yhb183264796560823@163.com (H.Y.); zhouhanyu27@163.com (H.Z.); lyg236200@163.com (Y.L.); fanxie1998@163.com (F.X.); 15656098967@163.com (Q.W.); 2 Anhui Provincial Key Laboratory of Livestock and Poultry Product Safety Engineering, Institute of Animal Husbandry and Veterinary Medicine, Anhui Academy of Agricultural Sciences, Hefei 230031, China

**Keywords:** circRNA, resveratrol, apoptosis, ovary, porcine

## Abstract

Circular RNAs (circRNAs) are a class of circular non-coding RNAs that play essential roles in the intricate and dynamic networks governing cell growth, development, and apoptosis. Resveratrol (RSV), a non-flavonoid polyphenol, is known to participate in follicular development and ovulation. In our previous research, we established a model using porcine ovarian granulosa cells (POGCs) treated with resveratrol, which confirmed its regulatory effects on long non-coding RNAs (lncRNAs) and microRNAs (miRNAs) within these cells. However, the influence of resveratrol on circRNA expression has not been thoroughly investigated. To explore how resveratrol affects circRNA levels in POGCs, we designed an experiment with three groups: a control group (CON, n = 3, 0 μM RSV), a low-dose RSV group (LOW, n = 3, 50 μM RSV), and a high-dose RSV group (HIGH, n = 3, 100 μM RSV) for circRNA sequencing. We identified a total of 10,045 candidate circRNAs from POGCs treated with different concentrations of resveratrol (0, 50, and 100 μM). Differential expression analysis indicated that 96 circRNAs were significantly altered in the LOW vs. CON group, while 109 circRNAs showed significant changes in the HIGH vs. CON group. These circRNAs were notably enriched in biological processes associated with cell metabolism, apoptosis, and oxidative stress. Functional enrichment analysis of the host genes revealed their involvement in critical signaling pathways, including mTOR, AMPK, and apoptosis pathways. Additionally, we identified potential miRNA sponge candidates among the differentially expressed circRNAs, particularly novel_circ_0012954 and novel_circ_0004762, which exhibited strong connectivity within miRNA-target networks. Our findings provide valuable insights into the regulatory mechanisms of circRNAs in the context of resveratrol-induced apoptosis in POGCs, highlighting their potential as innovative therapeutic targets in reproductive biology.

## 1. Introduction

The ovary, which secretes hormones, produces oocytes, and provides an environment for fertilization, implantation, or pregnancy, is crucial in the reproductive biology of female mammals [1]. Follicular atresia is a common natural physiological phenomenon in mammals. During an estrous cycle, less than 1% of follicles mature and ovulate, while the majority of the rest undergo atresia [2]. The proliferation and apoptosis of ovarian granulosa cells are pivotal in the process of follicular atresia [3]. Therefore, elucidating the molecular regulatory mechanisms of follicular atresia and ovarian granulosa cell apoptosis is of great significance for understanding the role of mammalian ovarian function and improving ovulation rate.

Resveratrol (RSV), also known as 3,4,5-trihydroxystilbene, is a polyphenolic compound recognized for its potent antioxidant properties. It has been identified in a variety of sources, including grapes, peanuts, and over 70 other plant species [4]. Studies have shown that resveratrol has a potential role in reproductive regulation in mammals [5]. For instance, resveratrol can improve ovarian failure and normalize the estrus cycle in rats [6]. Additionally, it has been observed to suppress DNA synthesis within the theca-interstitial cells of the ovary in vitro [7] and can also inhibit steroid production by inhibiting the expression of Cyp17a1 [8]. There are some interesting phenomena in ovarian granulosa cells. Resveratrol induces a biphasic impact on DNA synthesis [9], with lower doses promoting the uptake of thymidine and higher doses leading to a reduction in its incorporation. This similar phenomenon has also been confirmed in other studies. Resveratrol reduces estrogen production in rats in a manner that is dependent on its concentration, while it does not notably impact the synthesis of progesterone [10]. In experimental studies, resveratrol has a significant effect on ovarian morphology (improving follicle development) and improving the estrus cycle [11]. Similarly, resveratrol can reduce the spindle deficiency of porcine oocytes and avoid their aging in vitro [12]. These findings suggest that resveratrol may effectively regulate the ovulation process of follicles.

Circular RNAs (circRNAs) are a novel type of non-coding RNA with diverse functions. Circular RNAs are created through a splicing process that joins exons and introns, forming a circular conformation. This unique structure sets them apart from linear RNAs, allowing them to evade degradation by nucleases [13]. A spectrum of circRNAs has been identified, including those derived from exons, intergenic regions, and introns [14], of which exon circRNAs account for the majority and are produced by the expression of known protein-coding genes. Reports indicate that circRNAs serve multiple roles, including the competitive inhibition of miRNAs and the direct modulation of the expression of their parent mRNAs [15,16], and play an important role in regulating the growth and development of primitive follicles in mammals [17], ovaries [18], and uterus [19]. For example, Xi et al. [20] identified 22 circRNAs as hub circRNAs on Xiang pig estrus, which may play a potential role of circRNA in Xiang pig ovary regulation. According to Liu et al. [21], it was revealed that circRNAs play a key role in the prolificacy trait and the transition from the follicular to the luteal phase during the estrous cycle in goats. Pan et al. [22] characterized the ovarian circRNA profiles during pubertal transition in gilts and found the biological functions of parental genes of circRNAs were enriched in progesterone-mediated oocyte maturation and the steroid biosynthesis signaling pathway.

In our previously published studies, resveratrol has been shown to regulate the expression of lncRNA [23] and miRNA [24] during the apoptosis of porcine ovarian granule cells, and some important candidate non-coding RNAs have been mined. However, how RSV regulates the expression of circRNA and thus participates in the regulation of porcine ovarian granule cell apoptosis is unknown. Thus, this study mainly focused on identifying the specific circRNAs for the apoptosis of porcine ovarian granulosa cells by RSV treatment, with the aim of exploring the role of RSV in containing circRNAs in order to better understand the new candidate regulatory circRNAs for the apoptosis of porcine ovarian granulosa cells and to reveal new candidate regulatory circRNAs underlying the apoptosis of porcine ovarian granulosa cells. The circRNA expression profiles in porcine ovarian granulosa cells exposed to varying concentrations of RSV were investigated. GO (Gene Ontology) and KEGG (Kyoto Encyclopedia of Genes and Genomes) analyses were performed to reveal several biological processes of the host genes of circRNAs influenced by RSV, and some potential functional circRNA-miRNA interaction networks have also been identified. These screening data enrich the research on the regulation of apoptosis of porcine ovarian granulosa cells by RSV and provide a reference for the application of RSV in animal husbandry.

## 2. Materials and Methods

### 2.1. Ethics Approval

The animal use protocol listed below has been reviewed and approved by the Institutional Animal Care and Use Committee (IACUC), Anhui Agriculture University, under permit no. AHAU20201025.

### 2.2. Culture POGCs Treated with RSV

Fresh pig ovaries were obtained from Landrace gilts in the follicular phase at a local slaughterhouse located in Hefei, Anhui, China. The gilts had an average weight of approximately 150 kg and were approximately 1 year old. Vibrantly colored ovaries with abundant antral follicles and fullness were selected. To aspirate the follicular fluid without causing vessel disruption, small, healthy follicles ranging from 3 to 5 mm in diameter characterized by pink, well-vascularized walls were punctured using a disposable syringe. To mitigate individual animal variations, transparent follicular fluid was pooled from over 50 gilts’ ovaries. The fluid was centrifuged at 1200× *g* for 5 min, then resuspended and subjected to another round of centrifugation. The pellet containing pig ovarian granulosa cells was promptly seeded in 6-well plates at a density of 106 viable cells per well and cultured. The cells were cultivated in Dulbecco’s Modified Eagle Medium (DMEM), which was supplemented with 10% fetal bovine serum and a 1% antibiotic mixture of penicillin and streptomycin (Invitrogen, Carlsbad, CA, USA). This cultivation was carried out at a temperature of 37 °C under a humidified atmosphere comprising 5% CO_2_ and 95% air. When the porcine ovarian granulosa cells reached 80% confluency, the culture medium was aspirated. Subsequently, the cells were exposed to three different concentrations of RSV (0, 50, and 100 μM) obtained from Solarbio (Beijing, China) [23]. These concentrations represented the control, LOW, and HIGH groups, respectively. The porcine ovarian granulosa cells were subjected to triplicate cultures with the respective treatments for 24 h, following the aforementioned conditions. Following the cultivation period, the cells were collected, and the process of RNA extraction was carried out.

### 2.3. Library Preparation and Sequencing

Total RNA was isolated and purified from each sample using TRIzol reagent (Invitrogen, Carlsbad, CA, USA). The purity and integrity of the RNA were then detected using a NanoPhotometer^®^ spectrometer (IMPLEN, Westlake Village, CA, USA) and the Bioanalyzer 2100 system (Agilent Technologies, Santa Clara, CA, USA). RNA samples with an OD_260/280_ absorbance ratio ranging from 1.8 to 2.0 were selected for subsequent experimental procedures. A total of 5 μg RNA, isolated from each sample, was employed for the preparation of sequencing libraries. The total RNA was treated to eliminate ribosomal RNA, and linear RNA, allowing for the construction of strand-specific libraries. These libraries were generated by NEBNext^®^ UltraTM Directional RNA Library Prep Kit for Illumina^®^ (NEB, Ipswich, MA, USA) according to the manufacturer’s instructions.

Short RNA served as a template for the synthesis of the first strand of complementary DNA (cDNA) using a 6-base random primer. Then, a buffer, dNTPs (dUTP instead of dTTP), RNase H, and DNA polymerase I were added to synthesize the second strand of cDNA. To prepare the sequence-specific circRNA libraries, sequencing adapter sequence and EB buffer were added to the eluent for end repair, followed by a ligation reaction to generate templates for on-machine sequencing. Subsequently, each library underwent sequencing on the Illumina Hiseq 4000 platform.

### 2.4. Identification and Characterization of circRNAs

Through base recognition analysis, the raw image data files generated by the sequencing platform are processed and translated into the original sequencing reads. Subsequently, the original sequence data are processed to eliminate low-quality sequences and the adapter sequences required for sequencing in the internal Perl script to obtain clean data and calculate the GC content. The FA and GTF files for the reference genome and genetic model of the pig (*Sus scrofa*) are downloaded directly from the Internet (genome assembly: Sscrofa11.1 GCA_000003025.6). Clean data were compared with reference genomes and genes using Bowtie2 v2.2.8, and then circRNAs were detected and identified using find_circ and CIRI2.

### 2.5. Differential Expression Analysis of circRNAs

The original circRNA count obtained was normalized using TPM (Transcript per Kilobase per Million mapped reads) to represent the expression level of circRNA. After processing, the relative average TPM of each circRNA in the sample was obtained, and the DESeq R software package (1.10.1) was used to analyze the differences among each group. DE-circRNAs were screened under the condition of *p*-value < 0.05.

### 2.6. Function and Pathway Term Enrichment

In functional enrichment analysis, host genes of DE-circRNAs are mapped to each entry in the GO database, respectively. Hypergeometric tests are then conducted to determine which genes are significantly overrepresented in specific GO terms against the genomic backdrop. The GO analysis for these host genes is executed with the GOseq R package, which classifies gene functions into biological process (BP), cellular components (CC), and molecular function (MF). Furthermore, KOBAS software (v2.1.1, http://bioinfo.org/kobas, accessed on 16 December 2023) is utilized to statistically assess the enrichment of circRNA host genes. The GO and KEGG terms for *p* < 0.05 were defined as significantly enriched.

### 2.7. DE-circRNA with Potential as miRNA Sponge

Based on miRNA data previously published by us [24], miRanda was employed to detect miRNA binding sites within the exons of DE-circRNAs. The miRNA-circRNA network was constructed using Cytoscape (v3.8.2, https://cytoscape.org, accessed on 16 December 2023).

### 2.8. Verification of the Expression Changes in CircRNAs

To test the accuracy of the sequencing results, we randomly selected nine circRNAs for RT-qPCR verification. The primer pair was designed by PrimerSelect in DNAstar and synthesized by TsingkeBio (Beijing, China). The *GAPDH* housekeeping gene served as a reference for amplification. The primers utilized in this research are detailed in Table 1. Real-time quantitative PCR reactions were performed using the iTaq Universal SYBR Green Supermix Kit on the Bio-Rad CFX96 Real-Time Detection System (BioRad, Hercules, CA, USA), and each sample was repeated three times to ensure the accuracy of the quantitative results. The relative expression levels of circRNA were determined using 2^−ΔΔCt^.

### 2.9. Statistical Data Analysis

Data are presented as means ± standard deviation (SD) for at least triplicates. The GraphPad Prism (v 5.0) software (San Diego, CA, USA) was used to analyze the results of RT-qPCR and for graphing.

**Figure 1:**
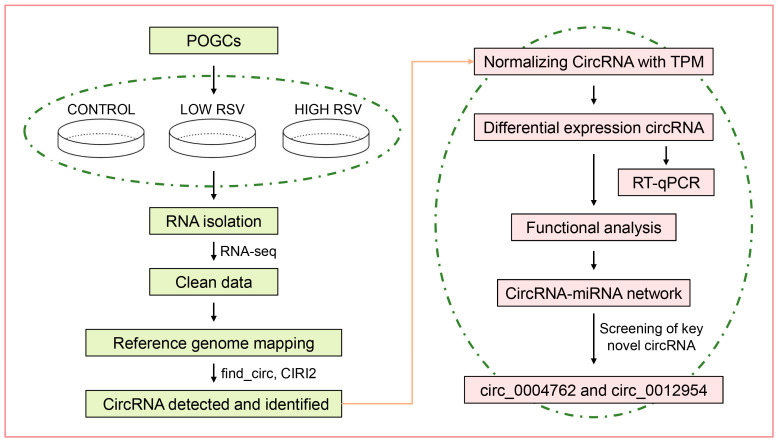
Workflow chart of sequencing data processing and verification.

For the above experiments, we carried out a workflow chart (Figure 1).

## 3. Results

### 3.1. Summary of circRNA Sequencing Results

To comprehensively detect circRNAs associated with resveratrol-induced apoptosis in porcine granulosa cells, we constructed nine libraries from three treatment groups. As shown in Table 2, in this study, the average amount of raw data obtained from each library was 92,084,199 (CON), 86,640,109 (LOW), and 88,926,401 (HIGH). With stringent filtering criteria, all libraries had a mapping rate of valid clean reads to the genome exceeding 84.07%. Furthermore, the quality scores (Q20 > 97.94%) and (Q30 > 94.00%) were achieved in all libraries. Furthermore, a total of 10,045 potential circRNA candidates were detected using the find_circ and CIRI2 tools (Table S1).

**Figure 2:**
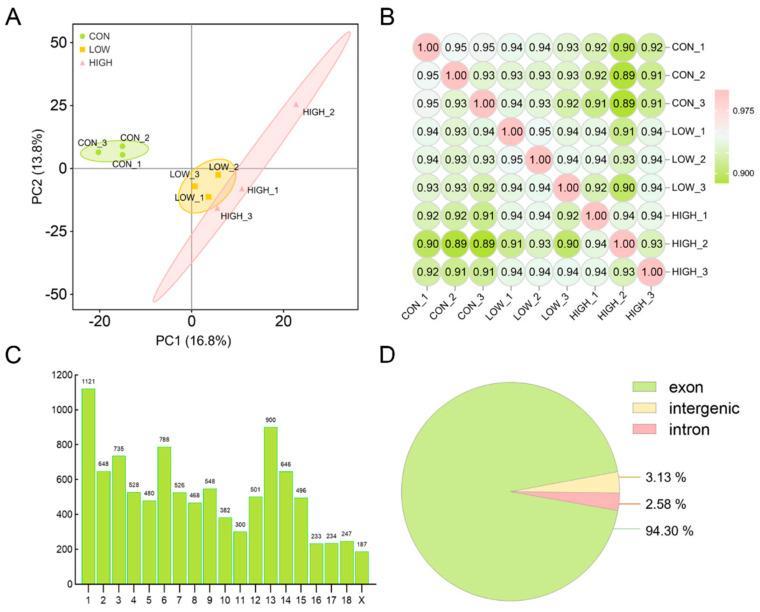
Characterization of candidate circRNAs in POGCs. (**A**) PCA of circRNAs in 9 POGC sam–ples. (**B**) Correlation heatmap. (**C**) The distribution of circRNAs on the *Sus scrofa* chromosomes. (**D**) The distribution of circRNAs in genomic regions.

Principal component analysis (PCA) of circRNAs revealed that three samples from each treatment group clustered together in porcine granulosa cells (Figure 2A). Sample correlation analysis demonstrated strong correlations within each treatment group (Figure 2B). Subsequently, the distribution of exons and chromosomes of circRNAs was analyzed, revealing a wide distribution of circRNAs across all chromosomes of the pig. Among them, 9781 circRNAs were transcribed unevenly from the 18 pairs of autosomes, while 187 circRNAs originated from the X chromosome. CircRNAs derived from chromosomes 1, and 13 exhibited higher abundance compared to any other chromosomes (Figure 2C). The majority of circRNAs (94.3%) were formed by exons, while 2.58% originated from introns and 3.13% from intergenic regions (Figure 2D). These findings confirmed the reliability of the sequencing data and characterized the features of circRNAs.

### 3.2. Differential Expression and Cluster Analysis of circRNAs

**Figure 3:**
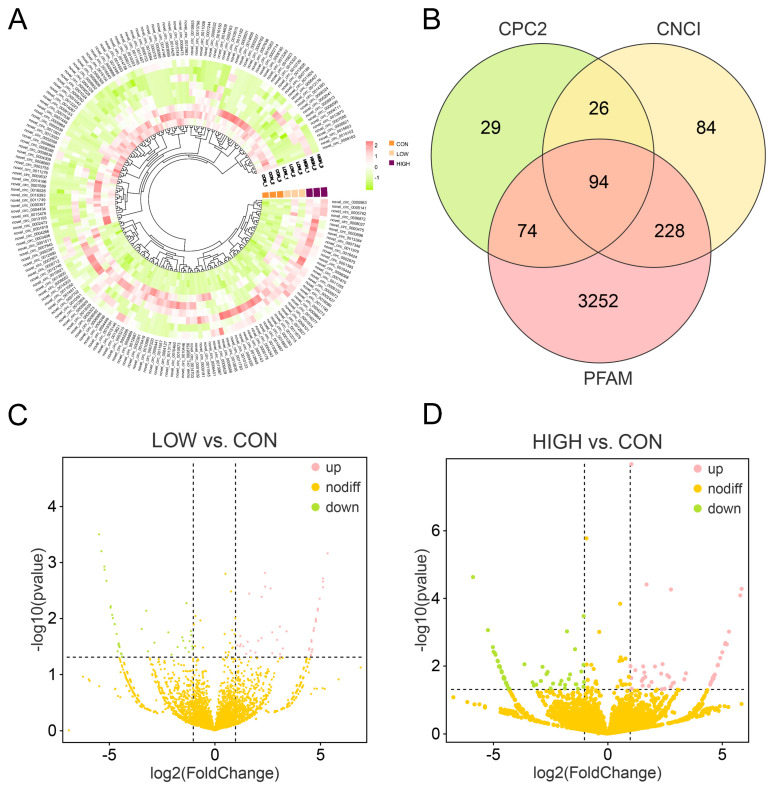
Expression analysis of circRNAs in POGCs. (**A**) Heatmap illustrating the relative expres–sion of DE-circRNAs. (**B**) Veen diagram of DE-circRNAs with coding potential. (**C**) Volcano plot of all DE-circRNAs between LOW vs. CON group. (**D**) Volcano plot of all DE-circRNAs between HIGH vs. CON group.

To better understand the regulation of circRNA in porcine granulosa cells, expression profiling of circRNAs identified in the CON, LOW, and HIGH groups was conducted. The heatmap (Figure 3A) illustrates the changes in all differentially expressed circRNAs (DE-circRNAs) identified in this experiment across the samples, and additionally, we used CPC2, CNCI, and PFAM to identify circRNAs with potential coding ability (Figure 3B). A total of 6338 circRNAs with an IRES element score > 0.5, indicating potential coding ability, were identified, and we found 94 circRNAs that were commonly recognized by the three databases as circRNAs with coding potential in this study. Furthermore, volcano plots were generated to illustrate the expression patterns of DE-circRNAs in the two pairwise comparisons (Figure 2D and Figure 3C, Table S2), revealing 96 DE-circRNAs (51 upregulated, 45 downregulated) in the LOW vs. CON group and 109 DE-circRNAs (47 upregulated, 62 downregulated) in the HIGH vs. CON group.

### 3.3. Confirmation of circRNA Expression by RT-qPCR

**Figure 4:**
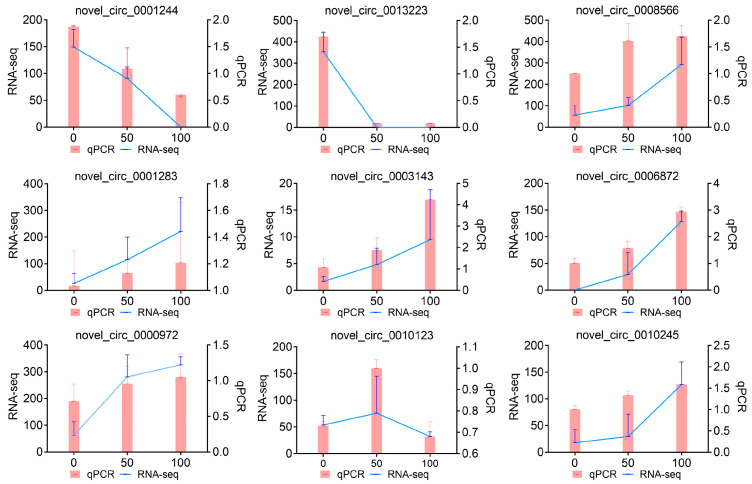
RT-qPCR verification of DE-circRNAs in POGCs treated with resveratrol. RT-qPCR (Bar chart, pink and circRNA-seq expression line chart, blue validation of the indicated POGCs circRNAs. The circRNAs expression levels were normalized to the *GAPDH* gene, and circRNAs expression was normalized to TPM.

Nine circRNAs were randomly selected from the novel circRNAs and their expression levels were measured by RT-qPCR at three groups (CON, LOW, and HIGH group). The results showed consistency between the RNA-seq and RT-qPCR data, indicating the reliability of the RNA-Seq results (Figure 4).

### 3.4. Functional Analysis of DE-circRNAs

**Figure 5:**
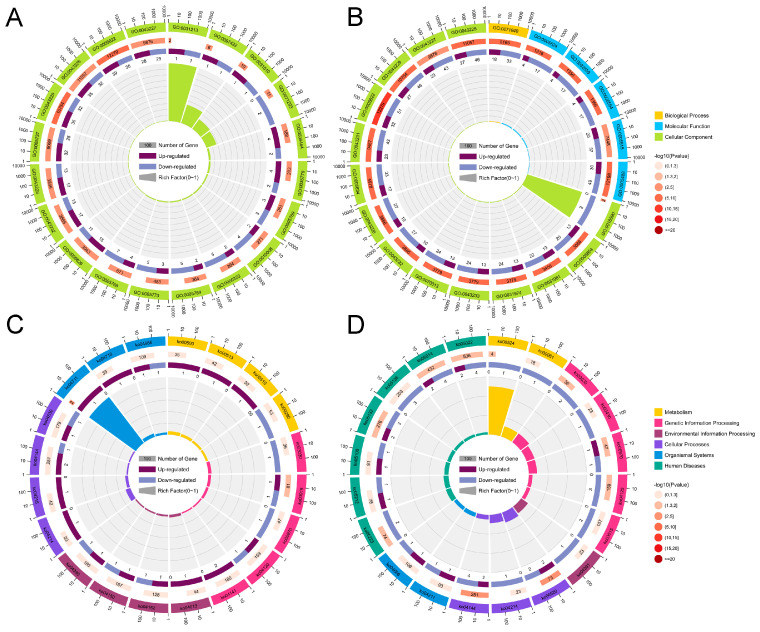
Function and pathway analyses of DE-circRNAs. (**A**,**B**). Top 20 GO terms of host genes of DE-circRNAs in the LOW vs. CON and HIGH vs. CON group, respectively. (**C**,**D**). Top 20 KEGG pathways of host genes of DE-circRNAs in the LOW vs. CON and HIGH vs. CON group, respec–tively.

To investigate the potential physiological and molecular roles of the host genes of (DE-circRNAs in the apoptosis of porcine ovarian granulosa cells induced by RSV, we perform GO and KEGG pathway enrichment analysis of host genes (Table S3). In LOW vs. CON group, the host genes of 96 DE-circRNAs were annotated with categories of 1901 BP, 327 MF, and 286 CC, and 447, 81, and 97 GO terms were significantly enriched in the BP, MF, and CC, respectively (Table S3A). The twenty most significant GO terms were shown in Figure 5A. Notably, host genes of DE-circRNAs were found to be primarily involved in cell activity, including endocytic recycling, cellular macromolecule catabolic process, negative regulation of translation, cellular catabolic process, response to redox state, and cellular protein catabolic process in biological process; protein binding, GTPase regulator activity, epidermal growth factor receptor binding, and estrogen receptor binding in molecular function. KEGG pathway enrichment analysis showed that the host genes were enriched in 82 pathways (Table S3B). Although most of the signaling pathways were not significant, some interesting pathways are still found in the top 20 pathways, including the RNA degradation, Endocytosis, AMPK signaling pathway, Circadian rhythm, Ubiquitin-mediated proteolysis, DNA replication, mTOR signaling pathway, and Hippo signaling pathway (Figure 5B). In the HIGH vs. CON group, the host genes of 109 DE-circRNAs were annotated with 2584 GO terms, which revealed that 316, 115, and 95 GO terms were significantly enriched in the BP, MF, and CC, respectively (Figure 5C, Table S3C). Among them, host genes are mainly enriched in cell apoptosis and metabolic process, including regulation of chromatin organization, protein phosphorylation, DNA replication, cellular aromatic compound metabolic process, and regulation of the extrinsic apoptotic signaling pathway. Furthermore, binding also appears to be a highly enriched category, such as ATP binding, adenyl ribonucleotide binding, protein binding, protein kinase activity, kinase activity, GTPase binding, and transforming growth factor beta binding. In KEGG pathway enrichment analysis (Figure 5D, Table S3D), Adherens junction, Endocytosis, DNA replication, Ubiquitin-mediated proteolysis were significantly enriched.

### 3.5. DE-circRNAs with Potential as miRNA Sponge in POGCs

**Figure 6:**
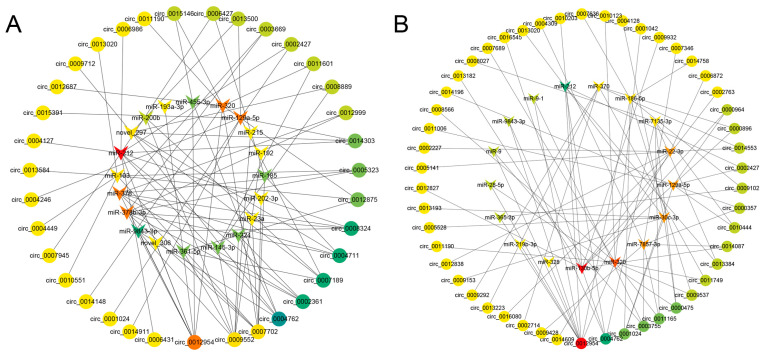
The circRNA-miRNA networks of LOW vs. CON (**A**) and HIGH vs. CON group (**B**).

To explore the potential role of circRNAs as miRNA sponges in pig ovarian granulosa cells, we employed the miRanda algorithm to predict the binding sites of miRNAs on DE-circRNAs. In the LOW vs. CON group, all DE-circRNAs targeted a total of 164 miRNAs, while in the HIGH vs. CON group, we identified binding sites for 184 miRNAs. Subsequently, we visualized the targeting relationships using Cytoscape software (v3.8.2) and selected the Top 10 nodes based on degree centrality through network analysis and the cytoHub plugin (Table S4). Figure 6A shows that in the LOW vs. CON group, the top five DE-circRNA nodes, namely novel_circ_0012954, novel_circ_0009552, novel_circ_0007702, novel_circ_0004762, and novel_circ_0002361, targeted at least four miRNAs each. In the HIGH vs. CON group, the top five DE-circRNA nodes, namely novel_circ_0012954, novel_circ_0004762, novel_circ_0001024, novel_circ_0003755, and novel_circ_0011165, targeted at least three miRNAs each (Figure 6B). These DE-circRNAs are considered to play crucial roles in both networks. Interestingly, novel_circ_0012954 and novel_circ_0004762 exhibit high degrees and target a larger number of miRNAs in both networks. Hence, these circRNAs were chosen as the most promising candidates for acting as miRNA sponges in the context of RSV-induced apoptosis in porcine ovarian granulosa cells.

## 4. Discussion

Granulosa cells are crucial in the processes of ovarian development and follicular atresia [25,26]. The proliferation and apoptosis of granulosa cells are physiological processes that occur in the normal female reproductive system [27]. These processes are regulated by hormone levels and cellular factors. In the study of follicular atresia, a large amount of evidence has been confirmed regarding the apoptosis of granulosa cells in various species, including pigs [28,29], poultry [30,31], and rodents [32,33]. RSV functions not only as a low-concentration natural antioxidant but also as a pro-oxidant that can increase cell apoptosis [34,35,36]. In our previous studies [23,24], we found that natural plant polyphenol resveratrol promoted the apoptosis of porcine ovarian granulosa cells. Additionally, we investigated the effect of resveratrol on the miRNA and lncRNA profiles of porcine ovarian granulosa cells. In the current study, we aim to explore how resveratrol influences the expression patterns of circular RNAs within porcine ovarian granulosa cells.

CircRNAs are a class of non-coding RNAs that are produced by splicing and participate in a variety of biological processes, including protein chelation, enhancement of parental gene expression, and transformation of peptides [37]. Existing studies have reported that circRNAs are divided into three categories: exon, intergenic, and intron. Exon circRNAs make up the majority of circRNAs, as indicated by previous research [38]. In our study, we have also found that exon circRNAs constitute approximately 94.30% of the total. Additionally, our findings show a broad distribution of circRNAs on chromosomes 1 and 13, which aligns with previous literature [22,38].

In addition, we identified 96 DE-circRNAs (LOW vs. CON) and 109 DE-circRNAs (HIGH vs. CON) in porcine ovarian granulosa cells treated with different concentrations of RSV. Drawing from these results, we postulate that the parental genes of differentially expressed circRNAs may influence various biological processes in porcine ovarian granulosa cells. Consequently, we proceeded to perform GO functional enrichment and KEGG pathway analysis of the host genes of DE-circRNAs. In our study, we observed that resveratrol-induced expression of circRNAs in porcine granulosa cells can participate in a wide range of cellular activities, including cell metabolism, apoptosis, estrogen receptor binding, and oxidative stress. These findings are consistent with some previous studies. Zhao et al. [39] analyzed the differential circRNA expression in Yili geese at different egg-laying stages and observed that the host genes of these circRNAs were associated with signal transduction, metabolism. Fan et al. [40] have indicated that circ_BECN1 can stimulate the proliferation of ovarian granulosa cells and advance cell cycle progression while also diminishing cell apoptosis by modulating the miR-619-5p/Rab5b regulatory axis. In another study by Yu et al. [41], they delved into the characteristics of the follicular fluid exosomal circRNAs in PCOS patients and pointed out that the host genes of DE-circRNAs were predominantly involved in lipoprotein granule receptor activity and the cholesterol metabolism. Additionally, we discovered some interesting signaling pathways, such as circadian rhythm, mTOR, AMPK, Hippo, and cell apoptosis signaling pathways. Wang et al. [42] compared the circRNA expression profiles of hen granule cells under different light conditions. Their research revealed that the host genes were significantly enriched in ovarian steroid production, along with the activation of the MAPK and PI3K-Akt signaling pathways. In Xiang pig ovaries among diestrus and estrus stages, the host genes of DE-circRNAs were mainly related to the estrogen signaling pathway and circadian rhythm [20]. Liu et al. [43] have reported on studies concerning the mTOR (mammalian Target of Rapamycin) signaling pathway, suggesting that PHB2 interacts with ERβ to trigger autophagy in porcine ovarian granulosa cells via the phosphorylation of mTOR. This study investigated the role of DE-circRNAs in RSV-induced apoptosis of POGC cells, providing new insights into the involvement of circRNAs in ovarian granulosa cell apoptosis.

Despite our investigation into the potential functions of circRNA host genes, there has been a surge of interest among researchers in recent years in exploring the capacity of circRNAs to engage in biological processes as miRNA sponges or encoding peptides [44,45]. Therefore, the ability of DE-circRNAs to act as miRNA sponges and their encoding potential were analyzed. We found that 94 circRNAs were identified as having coding potential in the three databases, and the IRSE score of them was >0.5. Subsequently, the miRNA binding sites of DE-circRNAs were predicted. Novel_circ_0012954 and novel_circ_0004762 with the most potential as miRNA sponges in the porcine ovarian granulosa cells apoptosis induced by RSV. The interactions between circRNA and miRNA are complex processes, so these predicted results provide ideas for future research. Although this study provides valuable insights, we recognize its limitations, and future studies should explore the role of these circRNAs in different physiological states and their interactions with other non-coding RNAs and proteins.

## 5. Conclusions

Overall, this study sheds light on the effect of RSV on circRNA expression in porcine ovarian granulosa cells and explores the potential role of these circRNAs in apoptosis, metabolism, estrogen receptor binding, and oxidative stress. Notably, the identification of novel_circ_0012954 and novel_circ_0004762 as miRNA sponges with coding potential sheds new light on the intricate apoptotic functions of circRNAs.

## Figures and Tables

**Table 1:** RT-qPCR primers in the study.

circRNA Name	Sequence	Product Size (bp)
novel_circ_0001244	F: TGGATGGCATGGAGCTAACA R: AACTGGTGAATTTTCACAGCTACA	93
novel_circ_0013223	F: AGCGTCTAGACCCACAAGGT R: CAGAACCCCTCTTGGGGAGA	146
novel_circ_0008566	F: CCCGGGACCAGTGTGAATTT R: GGGGAAAAGTAGGAGGGCAG	138
novel_circ_0001283	F: CGCCATGTGCGTTGTCC R: GTCCGCCGGTCGTTGA	149
novel_circ_0003143	F: GCCCTGGCAGAGAGTTACATT R: CTCCGTCAGATCGTAACCGC	120
novel_circ_0006872	F: CGGTTGCTGGCTCAGCTT R: TCACGGCACCCCTGGT	148
novel_circ_0000972	F: GCTCCGACAGAGCTCATTAACA R: TTGATCCCATCGGAACTAGCC	122
novel_circ_0010123	F: GCCGAGCAATTGTAATGCGATA R: CCTGAAAACAATTAAAGTCGGACCA	133
novel_circ_0010245	F: CCAGAGCCAAGGGCAAACTT R: CCTGTGGTCTCCTGCATCTG	133
*GAPDH*	F: ATTCCACCCACGGCAAGTT R: TTTGATGTTGGCGGGATCT	110

Note: Sequence and reference were obtained from NCBI, https://www.ncbi.nlm.nih.gov (accessed on 21 August 2023).

**Table 2:** Summary of the oviductal circRNA sequencing data.

Sample Name	Raw Reads	Clean Reads	Mapped Pairs Reads	Mapping Ratio (%)	Q20 (%)	Q30 (%)
CON1	97,446,372	94,245,104	84,846,154	87.07	98.27	95.09
CON2	94,450,368	91,312,586	82,848,924	87.72	98.26	95.04
CON3	84,355,858	81,398,735	71,745,502	85.05	98.35	95.26
LOW1	79,346,130	76,149,202	68,165,722	85.91	98.04	94.27
LOW2	95,927,666	89,822,719	80,650,420	84.07	98.17	94.84
LOW3	84,646,530	81,366,838	73,444,890	86.77	97.94	94.00
HIGH1	83,261,846	80,305,163	71,967,026	86.43	98.35	95.30
HIGH2	78,782,640	75,427,672	68,462,566	86.90	98.18	94.79
HIGH3	104,734,716	101,157,409	91,671,120	87.53	98.30	95.15

## Data Availability

The datasets presented in this study can be found in online repositories. The names of the repository/repositories and accession number(s) can found in NCBI BioProject PRJNA854769.

## Supplementary Materials

The following supporting information can be downloaded at: https://www.mdpi.com/article/10.3390/cells13181571/s1, Table S1: Identifying potential circRNA candidates using find_circ and CIRI2 tools. Table S2: The expression patterns of DE-circRNAs in the two pairwise comparisons. Table S3: The GO and KEGG pathway enrichment analysis of host genes. Table S4: PPI Network and visual analysis with Cytoscape.
